# Accelerated Internationalization Among Inexperienced Digital Entrepreneurs: Toward a Holistic Entrepreneurial Decision-Making Model

**DOI:** 10.1007/s11575-022-00469-y

**Published:** 2022-05-20

**Authors:** Mika Gabrielsson, Markus Raatikainen, Saara Julkunen

**Affiliations:** University of Eastern Finland, P.O. Box 1627, 70211 Kuopio, Finland

**Keywords:** Effectuation and networking, Digital entrepreneurship, International experiential knowledge, International commitment decisions, Accelerated internationalization

## Abstract

This study explores accelerated internationalization among inexperienced digital entrepreneurs who lack resources such as prior experience, knowledge, and networks, which previous research regards as prerequisites for such growth. Following an in-depth qualitative research methodology, the findings reveal three theoretical mechanisms through which inexperienced entrepreneurs can make international commitment decisions with regard to the internationalization of their digital firms. The first is a novel mindset-based approach through which an entrepreneur can make an affective commitment to the international stakeholders within a digital community. Entrepreneurs do that by applying pull-based tools in digital communication to build interest among potential network contacts. The second mechanism is a means-based approach following effectuation logic resulting in an effectual form of commitment to international stakeholders in the digital community. The mechanism relies on applying push-based tools for digital communication to facilitate interactions with known network contacts. The third mechanism is continuance commitment to international business that entrepreneurs can foster over time in tandem with accumulated international experiential knowledge. This research provides an entrepreneurial decision-making model that extends effectuation theory and integrates it with extant research. The resulting holistic entrepreneurial decision-making model explains the accelerated internationalization of digital firms.

## Introduction

Digital technologies enable entrepreneurial activity that incorporates novel technology and creates new business ventures. Consequently, there has recently been additional room for accelerated internationalization, particularly during the COVID-19 pandemic (see e.g., Fraccastoro et al., 2021a). The current research perspective is that digitalization is changing entrepreneurs’ decision-making processes and outcomes such that they are becoming more fluid and feature goals that are less predefined than previously (Nambisan, 2017). Jiang and Tornikoski (2019) reported that inexperienced entrepreneurs face several forms of uncertainty, which they neither fully comprehend nor know how to address. Interestingly, the number of firms in several industries successfully achieving accelerated internationalization, despite their entrepreneurs being inexperienced, seems to be growing (Luostarinen & Gabrielsson, 2006; Rosenberg, 2018). The common feature of many of these entrepreneurs seems to be that they run digital ventures that successfully leverage networks (Bell & Loane, 2010). This study aims to extend extant research by investigating entrepreneurs’ decision-making in connection to the networking of digital firms during accelerated internationalization.

Prior research proposes that entrepreneurial decision-making logics, such as effectuation, can facilitate networking and mitigate the resource shortfall that can hinder young and inexperienced firms (e.g., Dew et al., 2011; Galkina & Chetty, 2015; Prashantham et al., 2019; Sarasvathy, 2001). A complementary perspective stems from an emerging digital entrepreneurship stream of research suggesting that different elements of digitalization – such as artifacts (e.g., mobile apps), platforms (e.g., Apple Appstore), and infrastructure (e.g., social media) – facilitate the decision-making and networking of entrepreneurs to potentially enable accelerated internationalization.

The empirical part of this study explores a globally renowned and successful digital entertainment company, to which we assign the pseudonym *Alpha*. Alpha has expanded from being a mobile-game developer to creating movies and being at the center of an extensive franchising business. The firm started as a university project and became a global industry leader in less than 15 years. The firm has faced many key events and made some great decisions throughout its existence despite its entrepreneurs having almost no prior international business experience or networks at the outset. Alpha’s success runs counter to the conventional wisdom on international business that holds entrepreneurs’ international experiential knowledge determines venture creation, growth, and foreign expansion (Eriksson et al., 1997; Kessler & Frank, 2009; Politis, 2005). Effectuation theory (Sarasvathy, 2001) does not explain accelerated internationalization by inexperienced entrepreneurs running digital ventures; albeit the extension of the theory to interpret networking behavior is beneficial (Galkina & Chetty, 2015; Prashantham et al., 2019). The digital entrepreneurship stream of research has not addressed exceptional internationalization behavior either (Nambisan, 2017), although digitalization does have an intuitive appeal to explain this exceptional behavior.

Based on an extensive literature review of these two streams, we note a research gap around examining entrepreneurs’ decision-making in connection to networking within the digital community during accelerated internationalization. The current research also addresses the calls for studies focusing on digital entrepreneurs involved in accelerated internationalization, covering the entire firm lifecycle from birth to maturity (Cavusgil & Knight, 2015; Coviello, 2015). Consequently, this study focuses on the following research question: How do entrepreneurs’ decision-making logic and networking within the digital community interact over the course of accelerated internationalization?

The methodological approach selected in the current study responds to calls in recent international business research suggesting that the available qualitative research could benefit from greater methodological transparency (e.g., Ji et al., 2018). Instead of the currently popular positivistic approach, we are guided by the philosophy of social constructionism (Berger & Luckmann, 1967; Burr, 1995; Ritchie et al., 2013), the ontological assumption of subjectivism, and the subjective epistemological assumption (Eriksson & Kovalainen, 2015), associated with the data of specific descriptions (Denzin & Lincoln, 2011; Ritchie et al., 2013), such as discourses, understood as comprising meaning frames (Edwards, 1997; Hall, 1997). Birkinshaw et al. (2011) suggested that the use of interpretative qualitative methods and techniques can provide a comprehensive understanding of different processes in international business research. When researchers open organizational processes with questions based on *how*, *who*, and *why*, qualitative methods can reveal individual and collective actions as they unfold over time (Doz, 2011).

We contribute to the earlier international entrepreneurship literature by extending the research on entrepreneurial decision-making into the digital networking context. More specifically, we frame our investigation with the literature of effectuation and digital entrepreneurship and also clarify the roles of *a global mindset*, *entrepreneurial means*, *commitment*, and *learning and knowledge* in the internationalization of inexperienced digital firms.

Accordingly, we propose a revised entrepreneurial decision-making model, advocating three decision-making alternatives applied by entrepreneurs to increase commitment within the digital community. The value of that contribution (see Corley & Gioia, 2011) lies in the new entrepreneurial decision-making model presented explaining accelerated internationalization. That explanation also makes a major contribution to international entrepreneurship research by unveiling entrepreneurial decision-making in the context of accelerated internationalization (Galkina & Chetty, 2015; Oviatt & McDougall, 1994; Prashantham et al., 2019; Sarasvathy, 2001). The explanation is particularly apposite regarding networking within the digital community. Furthermore, our model acknowledges the role of new digital environment and the emergence of digital entrepreneurship in accelerating internationalization, which has largely been ignored and may partly explain this behavior (Nambisan, 2017). Finally, the proposed framework contributes to research examining the maturing of firms undertaking accelerated internationalization (see also, Gabrielsson et al., 2014).

## Theoretical Review

Our conceptual foundation is based on a systematic literature search of the most relevant journals related to studies investigating effectuation, networking, and digital entrepreneurship. We used Reuter’s Web of Science database and EBSCOhost (multiple databases) to perform an advanced search within relevant journals publishing international entrepreneurship research, such as Entrepreneurship Theory and Practice, Journal of the Academy of Marketing Science, Journal of Business Research, Journal of Business Venturing, Journal of International Business Studies, Journal of International Entrepreneurship, Journal of Marketing, Journal of Marketing Research, Journal of World Business, Management International Review, and Small Business Economics. We considered articles published up until 2021. We searched for the keywords *internationalization*, *accelerated internationalization, effectuation, commitment,* and *digital entrepreneurship* appearing anywhere in the text (including the title, abstract, keywords, and main text). Interestingly, the literature reviewed revealed that there is one stream of research under label entrepreneurs’ decision-making logic that covers research dealing with use of effectuation logic and networking. Moreover, there is another stream of research that can be labeled as digital entrepreneurship, which provides insights into how entrepreneurial firms can internationalize within the digital community. However, there is no mention in the literature about how entrepreneurial decision-making logic and related networking applies to digital entrepreneurship in the context of accelerated internationalization. The following sections review earlier research and draw conclusions based on those studies; we present our positioning against the identified key articles in Table 1.

**Table 1:** Positioning our research against earlier research

Author/s	Entrepreneurial decision-making	Digital entrepreneurship
*Sarasvathy (*2001*): Qualitative method* Illustrates effectuation through case examples and experiments and examines the connections between effectuation and existing theories and empirical evidence	*X*	
*Galkina and Chetty (*2015*): Qualitative method* Builds a bridge between effectuation and the Uppsala model of internationalization and shows how SMEs follow a non-predictive logic in networking for international market entry	*X*	
*Engel et al., (*2017*): Conceptual paper* Challenges the planning and instrumental perspective of networking and theorizes effectual networking process of entrepreneurs under uncertainty	*X*	
*Magnani and Zucchella (*2019*): Qualitative method* Explores strategic actions available to cope with uncertainty in the internationalization strategy of entrepreneurial ventures, such as effectuation, and suggests that a global niche strategy can be successful in coping with uncertainty	*X*	
*Prashantham et al., (*2019*): Conceptual paper* Examines new venture internationalization speed by distinguishing effectuation and causation-based networking and conceptualizes their effects on the speed of initial entry, international scope, and international commitment	*X*	
*Kerr and Coviello (*2020*): Conceptual paper* Reconceptualizes effectuation as a multilevel and multi-theoretical phenomenon that drives networking and is dependent on networks; also shows the simultaneous and interactional use of effectuation and causation under changing market dynamism, uncertainty, and opportunity development	*X*	
*Nambisan (*2017*): Conceptual paper* Conceptualizes digital entrepreneurship as an uncertain entrepreneurial setting that involves dynamic and less bounded processes and predefined goals and is influenced by three distinct elements of digital technologies: digital artifacts, platforms, and infrastructure		*X*
*Elia et al., (*2020*): Qualitative method* Focuses on the impact of digital technologies and collaboration on the entrepreneurial process, and proposes a definition of a digital entrepreneurship ecosystem by highlighting the integrated digital-output and digital-environment perspectives		*X*
*Sahut et al., (*2019*): Conceptual paper* Studies digital entrepreneurship from a digital information processing perspective and shows that both micro- and systemic level approaches can be adopted when examining new venture creation, business models, processes, platforms, and the ecosystems of digital entrepreneurship		*X*
*Ghezzi and Cavallo (*2020*): Qualitative method* Builds a framework to connect business model innovation, lean start-up approaches, and agile development in the context of strategic agility and shows that lean start-up approaches can be utilized to enable business model innovation and digital entrepreneurship		*X*
*Our study: Qualitative method* This research considers inexperienced entrepreneurs who lack resources, such as prior international business experience and networks, and who apply effectuation and use digital networks to advance the firms’ internationalization. The findings show the importance of three alternative avenues to overcoming the lack of international experiential knowledge and enabling international commitment among the digital community	*X*	*X*

### Entrepreneurial Decision-Making

Earlier research suggests that international experiential knowledge exerts a key influence on accelerated internationalization, such that prior knowledge of foreign markets can enhance the speed with which an entrepreneurial team perceives opportunities (Oviatt & McDougall, 2005; Reuber & Fischer, 1997). Entrepreneurs are always at the heart of decisions and are also the key individuals from a learning perspective. Penrose (1966) asserted.

Experiential knowledge can only be learned through personal experience. With experiential knowledge, emphasis is placed on the change in the services the human resources can supply which arises from their activity… experience itself can never be transmitted, it produces a change – frequently a subtle change – in individuals and cannot be separated from them (p. 53).

The balance between entrepreneurial resources (e.g., international experiential knowledge) and international market conditions (e.g., market turbulence and position in a network) can cause uncertainty for entrepreneurial firms expanding internationally relatively soon after their foundation (see, e.g., Magnani & Zucchella, 2019). While more information can address uncertainty (Luce & Raiffa, 1957), decision-makers never have full access to all available information (Kirzner, 1973). Hence, they are cognitively limited and likely to fail to solve highly complex problems in an objectively rational way (Williamson, 1981). Effectuation theory is useful in unpredictable situations, such as new business environments where decision-makers have opportunities to shape a future in which forecasting is difficult or even inconceivable (Gabrielsson & Gabrielsson, 2013; Jiang & Tornikoski, 2019; Sarasvathy, 2001).

The effectuation process differs from the traditional planning-based approach, which Sarasvathy et al. (2014) refer to as causation. An effectual entrepreneur focuses on the means available and determines what can be done (Sarasvathy et al., 2014). The entrepreneur’s background, identity, knowledge, and active networks trigger the adoption of an effectuation logic, which is then reflected in her/his decision-making process and actual behaviour. Sarasvathy (2008) proposed three questions to capture the process: *Who I am? What I know? Whom I know?* The answers to these three questions present the entrepreneur with the available means which are the starting point for the effectual process. For instance, entrepreneurs ask themselves, “What can I do?” and then engages with the stakeholders, resulting in their making *effectual commitments* to stakeholders. An effectual commitment is a means-oriented commitment with other stakeholders within the network which the entrepreneur makes in exchange for a voice in shaping some focal market they seek for their own benefit (Van Mumford & Zettinig, 2022). In addition, Sarasvathy’s effectuation theory describes the effectual entrepreneur adopting an affordable loss approach rather than one focusing on expected returns. This trial-and-error process allows the entrepreneur to experiment with potential business opportunities without risking bankruptcy (Sarasvathy, 2001, 2008).

Furthermore, effectual entrepreneurs must find committed partnerships that provide mutual benefits and new opportunities (Sarasvathy, 2001). Sarasvathy (2001) maintained that entrepreneurs who follow an effectual approach turn unexpected events into profitable opportunities that can lead to unexpected outcomes (Fisher, 2012). Rather than trying to analyze opportunities and avoid risks, effectual entrepreneurs control their future by focusing on shaping it through their own actions (Sarasvathy, 2008; Sarasvathy et al., 2014).

Some scholars have suggested that a global entrepreneurial mindset (“orientation to the world that shapes the behavior of the individual”, Rhinesmith, 1992, p. 63) and an immediate commitment decision are prerequisites of successful accelerated internationalization (Gabrielsson et al., 2008; Magnani & Zucchella, 2019; Oviatt & McDougall, 1994). In that case, the commitment precludes an experiential knowledge prerequisite and is affective. *Affective commitment* is triggered by another party identifying with the global perspectives of the entrepreneurs and the opportunities they discover or create during the internationalization process (Gabrielsson et al., 2008; Meyer & Allen, 1991).

Over time, new goals emerge from actions that can be pursued if resources are acquired. Having acquired new resources (e.g., international experiential knowledge, funding, or networks), entrepreneurs may amend their perceptions of their firm’s mission, vision, or strategy. The effectuation process may also become one based more on causal decision-making. In causation, the entrepreneur focuses on the target, which involves asking what should be done using underutilized network relationships and with partners that could confer benefits (Engel et al., 2017; Vissa & Bhagavatula, 2012). Causal decision-making typically requires experiential knowledge of market opportunities and associated problems. Having that knowledge can facilitate commitment decisions involving a fear of loss into the international digital market and its networks, best described as *continuance commitments* (Gabrielsson et al., 2008; Meyer & Allen, 1991). When a venture has acquired sufficient experiential knowledge of operating in a foreign market, it might consider new market-specific investments.

Effectuation theory has been advancing in huge leaps in conjunction with networking. Galkina and Chetty (2015) established that small and medium-sized enterprises (SMEs) utilize effectuation rather than causation to tackle the uncertainty of internationalization. The effectuation approach permits effectual commitments to network relationships, boosts the means available to their ventures and creates new opportunities. Coviello (2006), however, illustrated that intensifying international commitment through stronger and more strategically compatible international customer relationships may demand more planned networking (i.e., causation), and that a causally increased customer base and extended networks are likely to increase international revenues and learning in the case of an SME (Prashantham et al., 2019). Interestingly, Kerr and Coviello (2020) reconceptualized effectuation theory as a multilevel phenomenon that drives networking. The same research presented a model of how individual-level use of effectuation connects with dyadic interactions between people and mutual commitment, which ultimately explain entrepreneurial network dynamics. At this level, effectuation theory turns into an assessment of shared means and addresses the question “What can we do?” The answer can trigger changes to the structural and relational elements of a network.

Previous research suggests that entrepreneurial teams have an exceptional capacity for learning, which facilitates accelerated internationalization (Reuber & Fischer, 1997; Shrader et al., 2000). The age of a firm and the extent to which it is a knowledge intensive type are important for learning and rapid international growth (Autio et al., 2000; Puthusserry et al., 2020). Accordingly, young, knowledge-intensive firms enjoy the learning advantages of newness, which facilitate their learning about international expansion and help them avoid the barriers that impede learning among older firms. However, the assertion that new ventures benefit from moving immediately into international markets is controversial and does not seem to offer a satisfactory explanation of accelerated internationalization (Zhou & Wu, 2014).

Their ability to learn from partnerships can partially explain the accelerated internationalization of inexperienced firms (Mansoori & Lackéus, 2019; Read et al., 2016). Recently, Puthusserry et al. (2020) reported that SMEs utilize both internal and external sources of knowledge and apply various learning approaches, such as self-learning, trial-and-error, and experience during post-entry growth. Causation is particularly associated with information-based strategic responses (Van Werven et al., 2015; Vissa & Bhagavatula, 2012; Wiltbank et al., 2006, 2009), whereas effectuation is defined as a logic of entrepreneurial expertise that is dynamic and interactive – it creates new market opportunities and consists of two parallel cycles of acquiring new means and setting new goals (Fisher, 2012; Sarasvathy, 2008).

### Digital Entrepreneurship

Digital entrepreneurship refers to new venture creation and the transformation of existing businesses through the development of new digital technologies and/or the novel application of such technologies (European Commission, 2015). A prominent researcher in this field, Nambisan (2017), argues that digital technologies (e.g., mobile computing, data analytics, social media, cloud computing, blockchain encryption, or crowdfunding) shape entrepreneurial processes and outcomes to make them less bounded and predefined. Therefore, the question of interest is whether digital entrepreneurship provides tools that help understand accelerated internationalization by inexperienced entrepreneurs in today’s digitalized economy, which is more dynamic and uncertain than ever before.

Digital entrepreneurship research lies at the intersection of entrepreneurship and digital technologies and addresses the implications of digitalization on entrepreneurial processes and outcomes (Nambisan, 2017). Digital entrepreneurship is enabled by three distinct but related elements of digital technology that might facilitate internationalization; digital artifacts, platforms, and infrastructures (Nambisan, 2017; Nzembayie et al., 2019). Digital artifacts are defined as applications or digital or media components that are part of new products or services and provide end-users with a particular functionality or value (Kallinikos et al., 2013; Nambisan, 2017; Nzembayie et al., 2019). For example, mobile apps, software, and media content are inherently borderless digital artifacts. Digital platforms are shared common sets of services and architectures that host complementary offerings, including digital artifacts (Elia et al., 2020; Nambisan, 2017; Parker et al., 2016). Digital marketplaces, such as Apple’s Appstore and the Google Play Store, are examples of digital platforms that provide instant international distribution channels for firms using those platforms as a complementor. Digital infrastructures are digital technology tools and systems that provide communication, collaboration, and/or computing capabilities to support digital entrepreneurship (Nambisan, 2017; Nzembayie et al., 2019). Cloud computing, social media, and data analytics are examples of digital infrastructures that facilitate international communication and collaboration.

Digital technologies can be an outcome of entrepreneurial processes but also of the environment (or context) where those processes are conducted (Elia et al., 2020). Accordingly, digital technologies connect entrepreneurial actors and provide a new environment for the entrepreneurial process and the emergence of digital communities, which together can form digital entrepreneurship ecosystems (Sahut et al., 2019). Ghezzi and Cavallo (2020) recently showed that digital startups in highly dynamic and unpredictable environments tend to commit to an ecosystem or network of partnerships to enhance their position and transfer value to their customers. Marketing communication incorporates two approaches: “push is targeted outbound communication originating from the marketer, while the pull is inbound communication that is initiated by the end-customer” (Unni & Harmon, 2007, p. 30). However, often this pull from end-customers has been created by more general type of marketing activities of the marketer. These, concepts may be important as earlier research examining the location specificity of social media communication has suggested that communication often is more general in the early phase of internationalization and becomes more targeted as the internationalization advances (Fraccastoro et al., 2021b). However, research on the role of digital technologies – such as digital artifacts, platforms, infrastructures, and the resulting digital entrepreneurship ecosystems – in enabling accelerated internationalization remains in its infancy.

## Methodology

The study required a research philosophy and methods that offered the opportunity to explore and understand the entrepreneurs’ decision-making logics, networking, and digital entrepreneurship in one accelerated internationalizing SME in a modern, technology-oriented industry. The study applied the philosophy of social constructionism (Berger & Luckmann, 1967; Burr, 1995; Ritchie et al., 2013), the ontological assumption of subjectivism, and the subjective epistemological assumption (Eriksson & Kovalainen, 2015, pp. 15–16) associated with the data of specific descriptions (Denzin & Lincoln, 2011; Ritchie et al., 2013), as presented in Table 2. These philosophical and methodological decisions produced unique insights into the complex phenomena of decision-making and networking among digital entrepreneurs involved in accelerated internationalization and the associated changes over time.

**Table 2:** Research philosophy as well as the ontological, epistemological, and methodological assumptions in the study

Social constructionism	Knowledge is produced by exploring and understanding the social world of the people being studied, and by focusing on their meanings and interpretations (Berger & Luckmann, 1967; Burr, 1995; Ritchie et al., 2013)
Ontological Assumption	Subjectivism. Reality is understood as the basis of perception and is different for every person. However, a conceptual understanding of reality can also be shared. Moreover, individuals’ perceptions may change over time and contexts (Eriksson & Kovalainen, 2015, p. 14)
Epistemological Assumption	Subjective epistemological view of knowledge. There is no access to the external world beyond our observations and interpretations (Eriksson & Kovalainen, 2015, p. 15)
Methodology	The methodological approach focuses on the richness of the discourses based on the meanings of the internationalization process held by the informants. The approach allows for a deeper exploration and understanding of theoretical constructs in relation to empirically complex and subjective phenomena (Haytko, 2004; Ritchie et al., 2013). Discourses can be understood as meaning frames (Edwards, 1997; Hall, 1997) that offer a common perspective on selected issues and phenomena, and they offer a lens through which to view changes in understanding over time. Furthermore, this approach helps deliver holistic and contextualized research findings (Hartley, 1994), which is particularly important when seeking to understand firm internationalization, being inevitably embedded within their own contexts. Moreover, the approach reveals emerging patterns in the interplay among constructs, which leads to important theoretical insights

### Empirical Context and Data Collection

The entrepreneurial behavior is studied within the context of a digital-based SME, here assigned the pseudonym Alpha. Alpha has undertaken accelerated internationalization and was selected as a case study because it operates in the game development industry – a highly technology-oriented field. In addition the internationalization was driven by inexperienced entrepreneurs.

Three university students keen to develop games founded Alpha in 2003. The students had won a game development contest that summer, which attracted the attention of established firms in the games development industry, leading to the creation of Alpha. Within three years, Alpha was drawing 25% of its total sales revenue from Europe, 50% from the United States, and 25% from Asia (including China, Korea, and Japan).

We conducted eight interviews applying the ontological, epistemological, and methodological assumptions of our study. The interviewees had been central to Alpha’s development and included the firm’s founders, chief executive officers (CEOs), a chief technical officer (CTO), and a chief marketing officer (CMO) (see Table 2). Additionally, a game industry expert was interviewed to provide an objective view of the industry and an external perspective on Alpha’s success. This interview was also used to confirm the data gathered from interviews with Alpha staff. Before engaging in the primary interviews, we invited two informants to participate in pilot interviews to refine the planned questions. All key informants were assigned pseudonyms to protect their identities.

We asked the participants to describe the entrepreneurs’ decision-making and networking associated with Alpha, from its founding to the present, focusing on the firm’s internationalization process and its development and maintenance. Each interview began with background questions before the interviewees were invited to provide an open-ended, chronological account of Alpha’s internationalization. We encouraged the key informants to describe their subjective perspectives (Haytko, 2004; Ritchie et al., 2013) regarding changes and key events. The research team sought to obtain a comprehensive understanding of the interviewees’ experiences and how they organized them (Polkinghorne, 1995). Previous research has established that influential and knowledgeable informants are the most reliable sources of information, particularly if they can recall important real-time events (Huber & Power, 1985; Kumar et al., 1993). The research team asked questions designed to acquire a holistic and detailed understanding of the phenomenon in question (Graebner, 2009; Lipton, 1977). Examples of the interview questions include: *What was your vision in the beginning? How would you describe the different events of the internationalization process? How would you describe the entrepreneur’s decision-making process with respect to internationalization? And please describe the associated digital and non-digital networking of the entrepreneurs during this process.*

This study used multiple data sources to avoid subject bias (Jick, 1979) and also gathered secondary data relating to Alpha’s lifecycle. A search of the firm’s websites, business publications, and annual reports resulted in the identification of 38 links related to the firm’s early history (2003–2009), 22 links related to the success of the Alpha brand (2009–2021), 28 links related to Alpha’s collaboration with global consumer brands (2014–2016), and 17 links related to the creation of a movie version of an Alpha game (2016–2017). The secondary data complemented the primary data, confirming Alpha’s key events and providing context for the interviews, including the changes in Alpha’s internationalization process over time. Having gathered the data, we used triangulation to ensure the validity of the interviews, cross-checked the primary interview data for consistency, and, when appropriate, compared them with the secondary data (Lupina-Wegener et al., 2015). The interviewees were contacted by telephone if clarification was required, a step calculated to increase the reliability and validity of the datasets by enhancing trustworthiness and credibility (Schwandt, 2001). Each interview was recorded and the transcripts sent to the interviewees for verification. There were no significant discrepancies that could not be explained by the iterative nature of the data collection. Table 3 describes the empirical interview data and the secondary data in detail.

**Table 3:** Empirical interview data and the secondary data in detail

Position, Firm (time period)	Title, International Business Experience	Length of Interview(s) (minutes)	Number of Transcript Pages	Time of Interview	Secondary data links confirming the primary data
CEO, Alpha, (2003–2015)	Founding CEO, no previous experience	99	19	January 2016	38 links in total related to the firm’s early history 22 links in total related to the success of the Alpha brand 28 links in total related to Alpha’s collaboration with global consumer brands
CMO, Alpha (2010–2016)	Head of marketing, traditional marketing experience	100 (pilot interview), 110	46	May 2013, January 2014	22 links in total related to the success of the Alpha brand 28 links in total related to Alpha’s collaboration with global consumer brands
CTO, Alpha (2003–present)	Founding CTO, no previous industry experience	75 (pilot interview), 100	32	May 2013, September 2013	38 links in total related to the firm’s early history 22 links in total related to the success of the Alpha brand
First external CEO, Alpha, (mid-2015–mid-2016)	Second CEO, traditional international marketing experience	50	9	June 2016	22 links in total related to the success of the Alpha brand 28 links in total related to Alpha’s collaboration with global consumer brands 17 links in total related to Alpha movie creation
Second external CEO, Alpha (2016–present)	Third CEO, lawyer, traditional firm experience	48	9	August 2016	22 links in total related to the success of the Alpha brand 28 links in total related to Alpha’s collaboration with global consumer brands 17 links in total related to Alpha movie creation
Game industry expert, Neogames (2004–2017)	External game industry expert, long industry experience	60	10	February 2015	22 links in total related to the success of the Alpha brand 28 links in total related to Alpha’s collaboration with global consumer brands 17 links in total related to Alpha movie creation

### Discourse Analysis

The deep, rich dataset encouraged us to find an appropriate method to explore the decision-making logic and digital entrepreneurship of the entrepreneurs at the heart of a digital accelerated internationalized SME. Therefore, we decided to focus on investigating the meanings (Riessman, 1993) embedded in the discourses of key informants from Alpha describing the firm’s internationalization process (Eriksson & Kovalainen, 2015; Hall, 1997). Because people describe how they think about a topic, event, or experience through discourse, this method allowed us to analyze the changes in meaning structures over time and, thus, to understand the interviewees’ decision-making processes and digital entrepreneurship during the firm’s internationalization process. One advantage of this approach is that the meanings found in informants’ discourses always follow the logic of their way of organizing and understanding real-life events and environments (Riessman, 1993).

Two researchers conducted the initial data analysis and carefully reviewed the data together to identify the key events in Alpha’s history; this process aligned with the recommendations of Lupina-Wegener et al. (2015). Next, each researcher continued their analysis individually to obtain a comprehensive overview of the key events. They explored the meanings by focusing on each key event and allowing changes in meaning to emerge inductively. Conducting two separate analyses and using the secondary data to support and strengthen the primary data helped the researchers interpret the key informants’ independent and shared meaning systems, which are socially constructed and understood as subjective (Burr, 1995; Haytko, 2004; Ritchie et al., 2013). In parallel with the meaning analysis, the researchers abductively discussed the relevant literature and their empirical interpretations to corroborate the most relevant insights (Kanitz et al., 2021).

Finally, the researchers compared their interpretations of the meanings and discussed any differences, resolving instances of disagreement through further discussion and data analysis, as suggested by Jick (1979). When they had developed a shared understanding, the researchers built the discourses together based on the meanings and followed a timeline of key events.

## Findings

Exploring the interaction between entrepreneurs’ decision-making logic and networking within the digital community during Alpha’s accelerated internationalization revealed two discourses: the discourse of international experiential knowledge and decision-making logic and the discourse of international commitment to digital communities. Both discourses were identified in the entrepreneurs’ descriptions of the meanings that changed over time. Specifically, the discourses illustrated a path to overcoming a lack of international experimental knowledge, showed an evolution of decision-making logic, and recognized the commitment to international digital communities during Alpha’s accelerated internationalization. Table 4 summarizes the key events in Alpha’s history and the discourses in meanings that revealed the firm’s path to internationalization. Representative interview excerpts are presented to clarify the findings on each key event.

**Table 4:** The key events in Alpha’s history and the discourses revealing Alpha’s path to internationalization

Key events	Discourse: International experiential knowledge and decision-making logic	Discourse: International commitment to digital communities
*2003 Assembly game development contest*	Meaning: Lack of international experiential knowledge, passion for global game development, and effectual decision-making	Meaning: Associating with the actors of the digital network
	Quotations: *We just wanted to pursue our own cool dream* [to be game developers globally]*.* (CMO) *Sometimes you just need to believe in what you’re doing and also dare to start despite uncertainty about the final result; you just have to try. (Founding CTO)*	Quotations: *There are a few influential studios and their executives, some influential publishers, some influential investors, and people like that. And if you know these people, you have access to a lot of places.* (Game industry expert) *Active networking* [in digital fairs and digital channels] *was something that we were able to do with the resources we had in hand.* (Former CEO, Rovio)
→Lack of international experiential knowledge, passion for global game development, and a global mindset-based effectuation approach (*Who I am; Dream to become global*) and talent in digital programming *(What I know)* leads to the founding of a new venture and generating interest through associating with the essential digital networks of game publishers (*How I can interest others; Creating pull in digital networks)* (Synthesis 1a). The Assembly game development contest also brought new contacts, including cooperation with a global game developer, Beta (*new means*) (Synthesis 2a)		
*2004 Cooperation with Beta*	Meaning: Capability in mobile-game development opened the door to effectual cooperation with a multinational game publisher in the digital industry	Meaning: Effectual commitment by a mutual game development collaboration with one MNC provides access to the international digital community
	Quotations: *We were very active… it’s more about going intuitively forward… they* [Beta] *offered global distribution, immediate potential at that volume.* (Former CEO, Rovio) *They [Beta] gave us a lot of autonomy… They didn’t want to control what we did too much. Which is, of course, was also an opportunity.* (Founding CTO)	Quotations: *In the beginning, we had to network aggressively* [using]*…Someone has to be enthusiastic and believe… So, they* [Beta’s management] *knew what they were doing, and it looked like they wanted our vision to turn into a game.* (Founding CTO) *The gaming industry is a people industry, the main thing is who you know. What you can do and who you know, because the business revolves around networks…* (Games industry expert)
→The cooperation with Beta was effectual and led to mutual effective commitment and access to the digital community, thus spurring accelerated internationalization (Synthesis 1b). However, over time, Alpha recognized it had to become more independent (*New Goals*) (Synthesis 2b)		
*2006 Alpha rebranded as Alpha Ltd*	Meaning: Initial international experiential knowledge about becoming overdependent on telecom operators and enhanced digital capability (as new means) in mobile-game development provided entrepreneurial means (strong self-efficacy) to follow an effectual approach	Meaning: Effectual commitment in global game development collaboration with several multinational corporations encourages the development of Alpha’s brand to enhance visibility with the end-users of the digital community
	Quotations: *I think it* [Alpha rebranding as Alpha Ltd. in 2006 with a focus on selling to operators] *can be summed up as, “It sucked!”* [laughs]*. It was a monopoly…the whole ecosystem…Or let’s say there wasn’t one, it was a tyranny. And they* [the operators] *abused their position and, at the same time, they were digging their own graves.* (Founding CTO) *We knew that there was this publisher that had by that time managed to get its games into the top ten [in the App Store]. We went to see them and asked if they wanted to publish this game… We thought that it would be more likely that we would get to Apple with them than without them. (CEO)*	Quotations: *We had to do subcontractor cases. We worked for all the big ones such as EA, Vivendi, Namco, Nokia, all of them… Finally,* *We were so deep in the hole that our wish was just to be able to make a game that would bring us enough financial success that we could achieve financial independence and earn a living from it, and make more games, and hopefully create a brand that would give us a living for a long time.* (CEO)
→Strong self-efficacy in international business and digital capability in game development *(What I know)* were utilized in a means-based effectuation approach providing confidence in bypassing the existing MNC controlled distribution system (*What I can do*) and *creation of push through the digital communication* by interacting with key players in the digital community (Synthesis 1b). Based on collaboration with key players in the digital community, Alpha recognized that they need to gain brand visibility in the consumers of the digital community *(New goals)*. (Synthesis 2b)		
*2009 Alpha mobile game release in App Store and Android Market*	Meaning: International experiential knowledge and digital capability in mobile-game development were significantly enhanced based on the learning from previous MNC collaboration and unsuccessful expansion in 2006 leading to use of causation logic	Meaning: Continuance commitment to develop a leading product for global end customers and a successful international business in the digital industry. Led to seeking network partners with strong relationships with Apple and Google
	Quotations: *They made a series of strategically correct decisions, which were in a way based on a good understanding of the industry, a good understanding of developmental stages, a long history and know-how, both on the business side and the developmental side. (Games industry expert)*	Quotations: *Our competence was in how to create the best mobile experiences from the final consumer’s perspective. We were making that game for eight months, analyzing all the hit mobile and web games very carefully, and trying to understand what it would take to make a hit game. It was a very analytical process. We tried to eliminate the role played by luck. (CMO)* *The whole App Store thing enabled global distribution for smaller players; we didn’t have to negotiate with the operators anymore. (Founding CTO)*
→Enhanced international experiential knowledge, new analytical skills, and specific global mobile-game industry understanding provided the basis for moving toward a causation-based strategic decision to commercialize the first mobile game (*Digital artifact)* for global consumers supporting innovative touch-screen technology into the emerging digital global marketplaces (*Digital platform)* (Synthesis 3). Based on the expansion through many digital platforms, the entrepreneurs started to learn of the options to leverage Alpha’s brand through its extension. As an outcome of their continuance commitment to the Alpha brand and mobile-game development, Alpha released an innovative mobile game as a breakthrough product in the App Store and Android Market, which resulted in a new way of playing mobile games		
*2014 Expansion to new industries*	Meaning: International experiential knowledge developed into analytical skills supporting strategic planning and causal decision-making	Meaning: Continuance commitment to relationships with global consumer brand firms
	Quotations: *I also saw that we must do it because we had created a mobile-game brand and game brands don’t last long enough…I wanted the Alpha brand to last 30, 40 years, and maybe longer. That [movie] was a must in order to secure a future for our brand and the firm.* (CEO)	Quotations: *We hired the top guys for the job…Now we have around 10,000 products on the market; we have deals with all the major retailers…Our brand has grown, and we think that we can do everything bigger; we can take our brand and go to any brand or retailer we want.* (CMO)
→Strong international experiential knowledge and planning skills led to targeting and committing to franchising collaboration with global consumer brands through which the firm could leverage its global digital brand in gaming to new industry segments. The strong digital brand also led to unexpected contacts from various fields interested in building a connection to digitalization and a gaming image in their products (*new means*) (Synthesis 4a). However, the new strategy was unsuccessful and led to the fragmentation in Alpha’s businesses and the risk of a diluted brand value and a recognition that the brand required strengthening (New goal) (Synthesis 4b)		
*2016–2020 The Alpha movies*	Meaning: Alpha recognizes the vulnerability of the brand owing to being reliant solely on mobile games, and that expansion to a new digital business area would be important, but they lacked international experience from new fields and therefore applied an effectual approach	Meaning: Effectual commitment to exploring new avenues for brand extension led to collaboration with a world-leading global film studio
	Quotations: *We do look at various paths in terms of where they might lead. And we think about whether we could start doing something new with them. But, on the other hand, we also need to have a focus. We cannot indefinitely walk those paths to their end to see where they lead. But I don’t think you should close any doors before you’ve had a good look inside.* (Second external CEO)	Quotations: *We first started by finding a partner who was also an agent in Los Angeles, and he helped us get an idea of what this [movie] business is about and what steps you have to take to get a good start…We were able to learn how the merchandising business works, and, in the end, we were able to create it ourselves…and it works well.* (CEO)
→Lacking international experiential knowledge in new industries led Alpha to explore digital brand extension avenues in the USA. Effectual networking helped identify the digital movie industry as a potential expansion area. Alpha began a collaboration with a leading global film studio and secured its commitment to developing a new movie based on Alpha’s digital competences and strong digital brand (*What I can do*). (Synthesis 1b)		

Below we have also summarized Alpha’s accelerated internationalization discourses to provide telling insights into the phenomena explored.

### From the Assembly Game Development Contest to Collaboration with Beta

The Alpha founders were university students whose lack of international experiential knowledge was compensated for by their favoring an effectuation approach. They had a dream of becoming global, well-respected talents in digital programming (*who I am*). Because of their self-learned skills as amateur talents in mobile-game development, they believed in themselves and wanted to show their competence (*what I know*). However, regarding their international experiential knowledge, the entrepreneurs lacked the resources, dense social networks, and previous international management and industry expertise to facilitate the process of establishing a new venture with an international orientation.

In 2003, their success at *Assembly*, an influential game development contest, inspired them to develop more games and drew increased interest from important network contacts. In particular, the advice and support of industry experts attending the Assembly event reinforced the entrepreneurs’ belief in their digital abilities as game developers. Furthermore, success in this initial game contest provided a foundation for what our data indicate entrepreneurs should initially ask themselves: *How can I interest others*? The entrepreneurs joined crucial digital networks for game publishers, contacted international customers, participated in digital fairs, and contributed to digital communities (e.g., initial internet-based mobile-game actors and LinkedIn). By these means, they attracted more interest, creating a pull factor in digital networks. As a result, the young entrepreneurs were recognized as new digital talents among game developers in the mobile gaming industry network, which encouraged them to establish a new venture.

Although the entrepreneurs lacked international experiential knowledge, their capabilities in mobile-game development and digital communication (e.g., internet-based communication via email, blogs, forums, and chats) facilitated access to Beta, a multinational corporation (MNC) digital games publisher, in 2004. Because Beta management had identified the entrepreneurs behind Alpha as potential partners, the young entrepreneurs made an effectual commitment to the MNC game developer (*new means*) and instigated close collaboration with them. This effectual commitment included an investment from the Alpha side into research and development work in order to meet Beta’s expectations. The collaboration set the Alpha entrepreneurs up with a new insider position and provided access to the global digital community, which spurred the firm’s accelerated internationalization. The Alpha staff described their relationship with Beta as active and beneficial, and Beta invested considerable resources into the partnership and, thus, shared the risk of the new venture. Although Beta gave Alpha significant autonomy in the collaboration, the Alpha entrepreneurs recognized the need to become more independent in their digital entrepreneurship.

### From Rebranding as Alpha Ltd. to the Launching of the Alpha Mobile Game

The Alpha entrepreneurs’ initial international experiential knowledge combined with the danger of overdependence and enhanced digital capability *(as new means)* in mobile-game development was the foundation of their self-efficacy. The perspective led to them adopting an effectual approach as a decision-making logic. Such entrepreneurial behavior enabled increasingly greater effectual commitment in global game development collaborations with several multinational corporations, and they felt able to bypass Beta, their MNC distributor. Alpha’s management created a push through digital communication (e.g., LinkedIn and other digital channels) by interacting with key players in the digital community – an example of employing effectual logic. Subsequently, the Alpha entrepreneurs committed to subcontract arrangements with several direct MNC customers in the digital mobile-game industry, which led them to invest the money received from an angel investor into rebranding the firm as Alpha Ltd. Such collaborations particularly helped the Alpha entrepreneurs recognize that they needed to gain brand visibility among consumers of the digital community *(as new goals)*. To reduce the firm’s dependence on any single relationship and to pursue rapid international growth, Alpha sold its own mobile games to operators, which triggered a staff expansion in 2006.

Owing to the entrepreneurs’ very limited experience of building a profitable business partnership, the expansion was unsuccessful. The entrepreneurs almost lost their financial independence and the firm failed to realize its international sales forecasts. However, Alpha’s entrepreneurs learned more about the game development industry and used that knowledge to develop new and better games. The entrepreneurs’ digital capability, which now included analytical skills and a specific understanding of the global mobile-game industry, prompted the move toward a causation-based strategic decision to commercialize their first mobile game (*a digital artifact).* The game deliberately targeted consumers globally and, in time, proved an enormous success. The innovative touch-screen technology exposed the game to new consumers in the digital mobile-game industry and was enabled by the emerging digital global marketplaces or *digital platforms*.

Alpha now had international experiential knowledge of how the digital mobile-game industry worked and what attracted the digital community. They used that knowledge and applied a causation decision-making logic during the development process of their new mobile game. The entrepreneurs understood that reaching the firm’s end customers relied on leveraging the marketing resources and digital platforms available to large multinational companies. Accordingly, Alpha adopted a causation-based process, seeking network partners with strong relationships with Apple and Google. As a result, a UK-based agency introduced them to Apple UK, which led to Alpha products being prominently displayed in Apple’s marketplace listings, first in the UK and then globally. In 2009, the entrepreneurs’ continuance commitment to the Alpha brand and mobile-game development led to Alpha’s innovative mobile game being marketed as a breakthrough product in the App Store and Android Market. The game changed the way mobile games were played. In the global mobile game market, the entrepreneurs understood the need to apply a different set of international experiential knowledge competencies, including calculation skills, to their global investments and partner selection.

### From the Expansion of New Industries to the Alpha Movie

The strong commitment of Alpha’s entrepreneurs to global digital business was now based on international experiential knowledge involving strong analytical skills to support strategic planning and causal decision-making. The Alpha entrepreneurs’ international experiential knowledge and analytical skills enabled them to form informed relationships involving continuance commitment with global consumer brand firms to leverage their global mobile-game brand to exploit new industry segments (new goals). Therefore, they targeted several franchising opportunities and quickly built partnerships with global consumer brands outside of the digital community. These new collaborations brought Alpha’s management team closer to the retail markets and pushed the firm toward new growth opportunities flowing from the expansion into highly diversified global consumer markets. The strong digital brand also led to unexpected contacts from various fields interested in associating their products with digitalization and gaming (new means).

However, the new strategy was unsuccessful and led to the fragmentation of Alpha’s businesses. The entrepreneurs’ extensive international experiential knowledge meant they recognized that their brand would deteriorate rapidly if Alpha relied only on leveraging the brand through international consumer firms. Consequently, the entrepreneurs began to explore developing a new digital business area. However, as they were unaware of the precise new direction it would take, the entrepreneurs applied a means-based approach. Having dispatched one of the entrepreneurs to the United States to explore possible avenues, the entrepreneurs used effectual networking to identify the digital movie industry as a potential area of expansion. They accordingly sought to collaborate with a leading global film studio and mutual commitments were made to develop a new movie based on the Alpha brand. The movie deal is an example of how a means-based effectuation approach (*what I can do*) can compensate for a lack of international experience in a new field. Alpha leveraged its strong brand to push through effectual commitments in new avenues to foster brand extension, and the entrepreneurs also formed an innovative collaboration with a world-leading global film studio. In May 2016, the computer-animated Alpha movie was released and enjoyed substantial worldwide success. This success was extremely important for the future of Alpha and its brand; it strengthened its global network position, and as a result, industry leaders sought to collaborate with Alpha. The discourses of the Alpha management informants reveal they view effectuation as an important decision-making tool that enables the firm to remain agile to exploit new digital business opportunities through innovative product development.

*The postscript.* In September 2017, Alpha entrepreneurs announced that the firm had collected 30 million euros through an initial public offering; the firm would subsequently be listed in 2017 on the NASDAQ stock exchange. Furthermore, by 2020, the Alpha entrepreneurs launched a second movie, and Alpha became recognized as a full-fledged global entertainment company.

## Discussion

### Synthesis and the Emerging Model

**Fig. 1:**
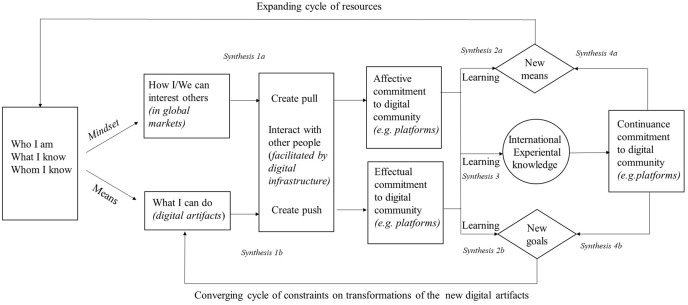
Entrepreneurial decision-making in digital firms during accelerated internationalization

This study examines how entrepreneurs’ decision-making logic and networking interact within the digital community over the course of an accelerated internationalization process. Prior effectuation research in a non-digital context has focused on entrepreneurs’ means-based approach, in which they begin with three categories of means: who I am, what I know, and whom I know (Sarasvathy, 2001, 2008). From a networking perspective, a central element in the means-based approach is the entrepreneurs’ question *whom I know* (Galkina & Chetty, 2015; Prashantham et al., 2019). However, digital entrepreneurs adopting the means-based approach to accelerated internationalization require prior international networks, which they do not necessarily have, as was the case in our empirical research. Hence, our investigation in the digital context expands current understanding by identifying two possible alternative entrepreneurial decision-making paths in effectuation: a novel mindset-based approach and a means-based approach. The paths reveal the changing nature of entrepreneurs’ decision-making and networking during accelerated internationalization. Figure 1 presents an emerging entrepreneurial decision-making and networking model reflecting accelerated internationalization within the digital community.

Within the mindset-based approach – a novel process revealed by our research – the entrepreneur mindset is driven by a global vision, which steers the search for new network contacts, initially with a focus on the question of *how can I/we interest others?* The aim is to obtain affective commitment within the digital community. For entrepreneurs lacking prior international experiential knowledge or networks, our empirical evidence suggests that utilizing pull-based digital communication channels can be effective. In that case, digital communications can reach a wide audience without relying on specific knowledge about network partners, with an objective to raise interest within a wider audience to make inbound connections. The tools involved are typically internet-enabled, such as forums and blogs, and can increase the visibility of a venture and actualize an affective commitment to stakeholders in the digital community.

The means-based approach initially focuses on *what I can do*. It begins with networking with existing contacts and moves to effectual commitment with the stakeholders in the digital community. Our empirical findings reveal that Alpha’s entrepreneurs were only able to adopt a means-based approach once they had acquired initial networks. When it is possible to adopt that process, it will reflect the original effectual model (Galkina & Chetty, 2015; Prashantham et al., 2019; Sarasvathy, 2001). However, our research suggests digital ventures employ means such as prior network contacts through a push-based approach making outbound connections to a targeted audience using, for example, emails, video negotiations, and interactive social media communication channels. Thereby, the digital communication targets known network partners, eventually leading to effectual commitment to the stakeholders in the digital community. Based on above discussion, we offer our first synthesis (See also Fig. 1).

*Synthesis 1: (a) During accelerated internationalization, entrepreneurs can follow (a) an initial global mindset-based approach leading to affective commitment, or (b) an entrepreneurial means-based approach leading to effectual commitment to the stakeholders within the digital community.*

The empirical evidence suggests that following the effectuation process by relying on either a mindset- or a means-based approach may involve adopting new means or setting new goals. A new finding pertaining to Sarasvathy’s (2001) model is that doing so requires harnessing learning and international experiential knowledge. Typical means are, for instance, a new network contact or new learned international experiential knowledge. For the Alpha entrepreneurs, their initial use of a mindset-based process resulted in a new network contact (the MNE Beta) that bolstered their initial resources (*Whom I know*) and enabled the subsequent means-based effectuation process.

In the next event, effectual decision-making resulted in the entrepreneurs acquiring new international experiential knowledge relating to the constraints of being dependent on a single partner. The situation persuaded Alpha to increase the number of partners involved in subsequent key events. This evidenced a converging cycle of constraints on the transformation of the new digital artifact that brought the firm back to adopting a means-based effectual approach and asking, “*What can I do.”* Hence, we offer the following synthesis:

*Synthesis 2: An entrepreneur that has established mutual commitment with the stakeholders within the digital community can embark on a repeated process of effectuation based on learning by following either (a) an expanding cycle of resources or (b) a converging cycle of constraints on the transformation of new digital artifacts.*

Recording the changes to the discourses over time enabled us to see beyond the initial choice between effectual decision-making and causal networking (Galkina & Chetty, 2015; Prashantham et al., 2019). The discourses revealed novel insights into the relation between learning and entrepreneurial decision-making, leading to different types of international commitments, including continuance commitment. Our findings indicate that the Alpha entrepreneurs’ effectual decision-making process and networking with the digital community generated learning. That learning, in turn, facilitated strategic causation-based decision-making and continuance commitments to international markets. This enabled the firm to make a huge investment in launching the main Alpha mobile game through digital platforms (Appstore, Google Play). Synthesis 3 below is intended to crystallize the changing process.

*Synthesis 3: During accelerated internationalization, the entrepreneurs will learn while engaging in effectual networking and accumulate international experiential knowledge that enables them to engage in causation-based decision-making when actualizing continuance commitment.*

Our findings also demonstrate that when the digital firm becomes mature, the entrepreneurs’ decision-making starts to resemble that of its larger and more established counterparts. The empirical evidence indicated that the entrepreneurs’ accumulated international experiential knowledge allows them to either adhere to an international experiential-based continuance commitment or return to effectuation. The latter course would demand committing to the expanding cycle of new means-based resources or the converging cycle of new goals necessary to overcome constraints to the transformation of new digital artifacts. Sticking with the new means-based approach was evident when the Alpha entrepreneurs started to expand into new industries through brand extension and the enhanced brand awareness attracted new (potential) partners. The new goals cycle was applied when the company realized that the strategy was depleting its brand value, forcing it to return to applying the effectuation process during its next iteration into film production. Therefore, our entrepreneurial decision-making and networking model of accelerated internationalization (see Fig. 1) extends earlier effectual networking research (Galkina & Chetty, 2015; Prashantham et al., 2019) by demonstrating the feedback loops available to a mature firm. Hence, we offer the following synthesis:

*Synthesis 4: As internationalization advances in a digital firm and the entrepreneurs engage in causal decision-making to attain continuance commitment, they may temporarily (a) return to effectuation by following the expanding cycle of resources or (b) the converging cycle of constraints on the transformation of the new digital artifacts.*

### Theoretical Contribution

The context for this study is that of entrepreneurs’ decision-making and networking within the digital community when engaging in accelerated internationalization, doing so successfully, despite lacking experiential knowledge. This phenomenon was investigated by focusing on two literature streams: entrepreneurial decision-making and digital entrepreneurship. An examination of qualitative research identified three entrepreneurial decision-making and networking paths for entrepreneurs to adopt in the course of accelerated internationalization (see Fig. 1).

This research contributes to the international entrepreneurship research field in the following ways. First, in terms of originality (Corley & Gioia, 2011), the study provides a holistic depiction of the entrepreneurial decision-making and networking paths available to digital entrepreneurs engaging in accelerated internationalization, an area on which earlier research has been silent. We thus extend effectuation theory (Read et al., 2016; Sarasvathy, 2001) and effectual networking research (Galkina & Chetty, 2015; Prashantham et al., 2019) by introducing the entrepreneurial mindset-based approach. That approach targets obtaining affectual commitment as an alternative to means-based effectual commitments. Our model (Fig. 1) also explains how entrepreneurs might engage in causal decision-making and the associated networking with increased continuance commitment; if they have accumulated international experiential knowledge.

Second, this study adds scientific utility (Corley & Gioia, 2011) to international entrepreneurship research. Explanations of the alternative means available to international entrepreneurs through digital networking remain underdeveloped (see e.g., Fraccastoro et al., 2021b). We present two alternative approaches: A pull-based digital communication approach that stimulates inbound contacts and eventual affective commitment among a wider audience without having specific knowledge of possible network partners; and a push-based approach in which digital communication is applied with specific network partners in mind.

Third, the current research incrementally extends the international entrepreneurship research field (Corley & Gioia, 2011): The developed model and the three associated mechanisms offer a solid basis on which researchers could build comprehensive qualitative and longitudinal research. Specifically, effectuation theory (Galkina & Atkova, 2020; Galkina & Chetty, 2015; Prashantham et al., 2019; Sarasvathy, 2001; Sarasvathy et al., 2014) and international new venture theory concerning accelerated internationalization (Oviatt & McDougall, 1994, 2005; Paul & Rosado-Serrano, 2019) could be extended to address what happens to the role of entrepreneurs as firms expand mature (Cavusgil & Knight, 2015; Gabrielsson et al., 2014). As internationalization advances, the decision-making may become more embedded at the firm level; however, our research indicates that the emphasis of decision-making may also start to alter between firm-level causal networking and entrepreneur-driven effectual networking.

## Managerial Implications, Limitations, and Future Recommendations

Following Corley and Gioia (2011), our findings can guide digital entrepreneurs acting as decision-makers and managers of accelerated internationalizing firms. The findings reveal how inexperienced entrepreneurs can choose from three different entrepreneurial decision-making and networking paths to achieve commitment within the digital community. The options depend on the entrepreneurs having the skills to use appropriate digital communication tools to expand their network contacts and achieve the necessary commitment among the digital actors. The findings also show that as resources expand, the form of decision-making absorbs more causal elements, and thus, inexperienced entrepreneurs’ decision-making comes to resemble that of their more experienced counterparts. However, our research emphasizes the importance of the entrepreneur cultivating organizational agility by retaining the option to implement entrepreneurial decision-making when a new opportunity calls for it. That agility is particularly important for digital entrepreneurs operating in an environment in a state of flux. Naturally, the current study – albeit offering a nuanced and processual understanding of a successful digital firm – suffers from the limitations specific to qualitative studies. Therefore, future longitudinal and processual qualitative research as well as quantitative panel studies that could provide more insights into the applicability of our proposed model and its generalizability to different contexts would be welcome.

Moreover, the entrepreneurial decision-making model for accelerated internationalization presented in our research could prove very useful in entrepreneurial education, particularly as there is a strong demand across society to promote digital entrepreneurship, as evident, for instance, in universities being expected to produce more science-based entrepreneurs, but there being few initiatives to ensure those often inexperienced graduates can select the most viable path to follow in their internationalization.

## Data Availability

Can be provided by the first author.
